# Impact of Age Stereotypes on Older Adults’ Cognitive Performance: An Experimental View on Aging Consumers

**DOI:** 10.1093/geroni/igaa057.2007

**Published:** 2020-12-16

**Authors:** Andrea Gröppel-Klein

**Affiliations:** University of Saarland, Saarbrücken, Saarland, Germany

## Abstract

The “contamination hypothesis” (Rothermund and Brandtstädter, 2003; Levy, 2003) assumes that negative external stereotypes significantly influence the cognitive and functional well-being of older people. Negative stereotypes also play an important role in consumer decision-making and responses to sales talks. Two surveys in home environments, using a snowball-system, with subjects randomly assigned to the different conditions in a 2x2-design (age stereotype x time pressure, study 1: n=151, Mage=65, study 2: n=122, Mage=68) show that older consumers, primed with negative age stereotypes, are less effective in correctly evaluating the value-for-money-ratios of different offers), especially when they perceive time pressure (=moderator). Self-efficacy is identified as a significant mediator, explaining the impact of stereotypes on performance. Contamination via “talking down” to older consumers also occurs in sales talks. In a “vignette” experiment, patronizing (vs. neutral) communication impairs the cognitive performance (measured via recall of information) of older consumers (n=86, Mage=69,) and leads to dissatisfaction.

